# Supplementary material for: Inhibition of DUSP18 impairs cholesterol biosynthesis and promotes anti-tumor immunity in colorectal cancer
Source: Nat Commun. 2024 Jul 12;15:5851. doi: 10.1038/s41467-024-50138-x (PMC11239938; doi:10.1038/s41467-024-50138-x)
Supplement: Supplementary file 1 — Supplementary Information [file 41467_2024_50138_MOESM1_ESM.pdf]

Supplementary Information

Inhibition of DUSP18 impairs cholesterol biosynthesis and promotes anti-tumor immunity in colorectal cancer

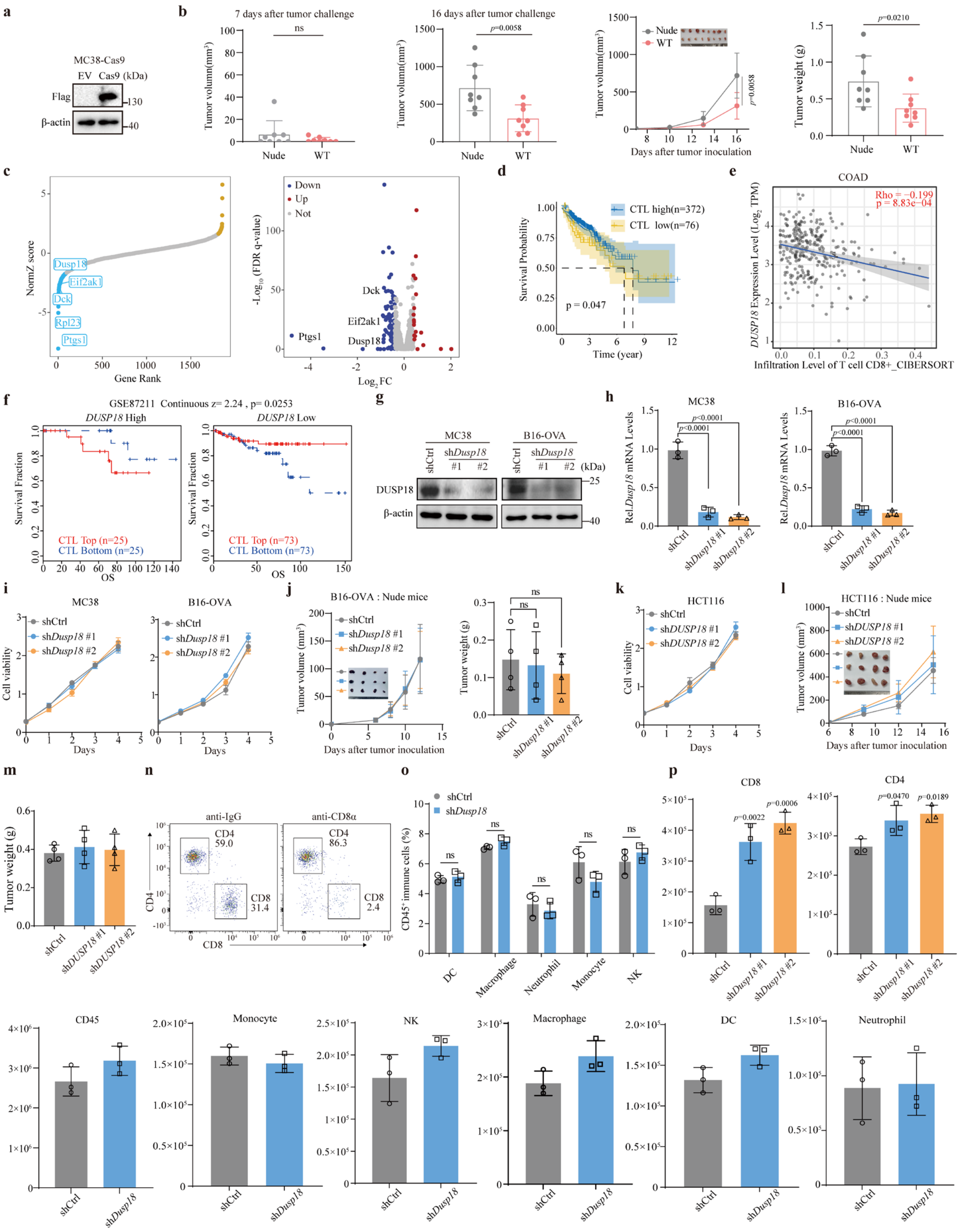

Supplementary Figure. 1 | The absence of DUSP18 enhances tumor-infiltrating CD8^{+} T cell function, related to Fig. 1. a, The stable expression of Cas9 was confirmed by Immunoblot (IB) analysis. **b**, Tumor volumes were measured 7 and 16 days after implantation in the drug targets, kinases and phosphatases library (KPD library) screens.n=8. Tumor growth curves and weights after implantation in the KPD screens. **c**, DrugZ-calculated NormZ score is plotted vs. gene rank for KPD screen (left) and DESeq2-calculated volcano map (right) showing sgRNA library screening distribution. Blue dots indicate significant deletion of sgRNAs. Red dots indicate significant

enrichment of sgRNAs (n = 2). **d**, Kaplan-Meier plot showing the association of cytotoxic T lymphocyte (CTL) scores (average expression of *CD8A*, *CD8B*, *GZMA*, *GZMB* and *PRF1*) with overall patient survival in TCGA COAD samples. Tumor samples were divided into two groups based on the median value of CTL scores. *P*-value was calculated by log-rank test. **e**, Correlation of *DUSP18* mRNA level and $CD8^+$ T cells infiltration in TCGA-COAD (n = 473). **f**, Association between cytotoxic T lymphocyte level (CTL) and overall survival (OS) for CRC patients (GSE87211 cohort) whose tumors were determined to be *DUSP18* high or low expression. Analyses were performed using Kaplan–Meier survival and Tumor Immune Dysfunction and Exclusion (TIDE) algorithm. **g, h,** Inhibition of *Dusp18* was validated by Immunoblot (IB) analysis (**g**) and qRT-PCR, n = 3 (**h**). **i**, Effect of *Dusp18* inhibition on proliferation of MC38 and B16-OVA cells (n = 3). **j**, 1×10^6 shCtrl or sh*Dusp18* B16-OVA cells were subcutaneously injected into nude mice (n = 4), tumor growth curves and tumor weight are shown. **k**, Effect of *DUSP18* inhibition on proliferation of HCT116 cells (n = 4). **l, m**, 1×10^6 shCtrl or sh*DUSP18* HCT116 cells were subcutaneously injected into nude mice (n = 4), tumor growth curves (**l**) and tumor weight (**m**) are shown. **n**, Flow cytometry analysis of $CD8^+$ and $CD4^+$ T cell subsets in splenocytes from anti-CD8a antibody-treated mice. **o**, Flow cytometric analysis of tumor infiltrating immune cells percentage after MC38 tumor cell inoculation. **p**, Immune cell numbers in MC38 tumors displayed in **Fig. 1n-p**, n = 3. Data are presented as mean ± SD (**b, c, h-p**). *P*-values were calculated by unpaired two-tailed t-tests (**b, c, p**), one-way ANOVA (**h-m, o-p**) or log-rank (Mantel-Cox) test (**d, f**). *P*-values and R were calculated by Spearman's correlation analysis. Two-sided *P*-value was given (**e**); ns, not significant. All IB data are representative of three independent experiments. Source data are provided as a Source Data file.

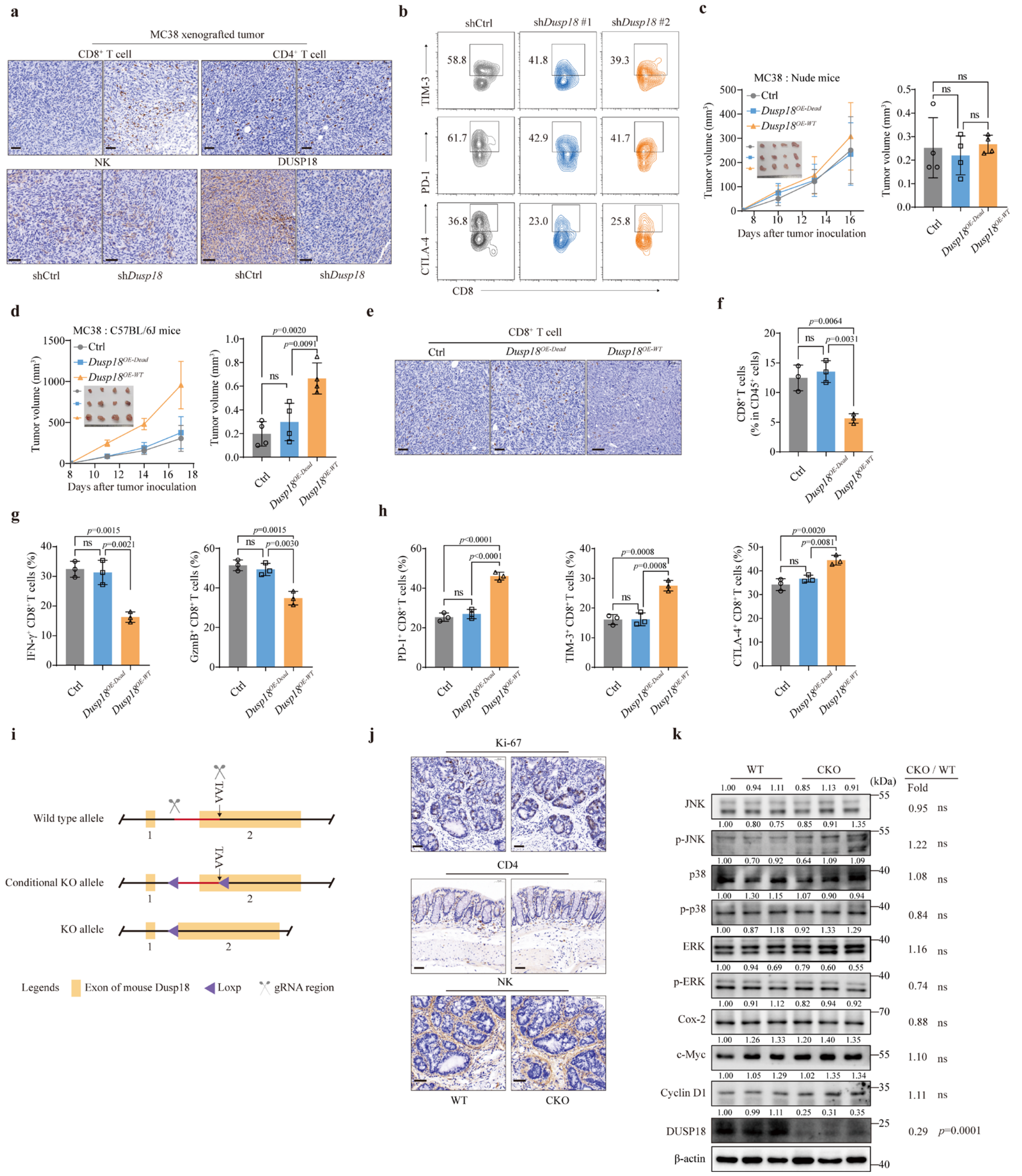

Supplementary Figure. 2 | Overexpression of *Dusp18*^{WT} in CRC cells inhibits tumor infiltrating CD8^{+} T cell function, related to Fig. 1. a, Immunohistochemistry showed the indicated cell types and protein level from MC38 tumors. Scale bar, 50 µm. The images are representative of 3 tumors. **b**, Quantification of PD-1, TIM-3 and CTLA-4 CD8^{+} TILs through flow cytometry. (n = 3) **c**, Tumor growth curve and tumor weight of the indicated groups in nude mice (n = 4). **d**, Tumor growth curve and tumor weight of the indicated groups in nude mice (n = 4). **e**, IHC staining of CD8 T cells in tumor tissues from the indicated groups as shown in (**d**) (left); Scale bar, 50 µm. The images are representative of 3 tumors. **f**, Quantification of CD8^{+} T cell infiltration through flow

cytometry (n = 3). **g**, Quantification of IFN-γ$^+$ and GzmB$^+$ CD8+ TILs. Tumor-infiltrating T cells were pre-stimulated with PMA, ionomycin and brefeldin A for 3 h (n = 3). IFN-γ and GzmB-producing cells was determined by flow cytometry. **h**, Quantification of PD-1, TIM-3 and CTLA-4 CD8$^+$ TILs through flow cytometry (n = 3). **i**, Schematic diagram for the construction of colon-specific deletion of *Dusp18* knockout mice. **j**, IHC showed the indicated cell types from AOM/DSS induced tumors. Scale bar, 50 µm. The images are representative of 3 tumors. **k**, Immunoblot (IB) analysis showed the indicated protein levels from AOM/DSS induced WT or CKO tumors. IB data are representative of two independent experiments. Source data are provided as a Source Data file. Data are presented as mean ± SD (**c, d, f-h**). *P*-values were calculated by one-way ANOVA (**c, d, f-h**), unpaired two-tailed t-tests (**k**); ns, not significant. Source data are provided as a Source Data file.

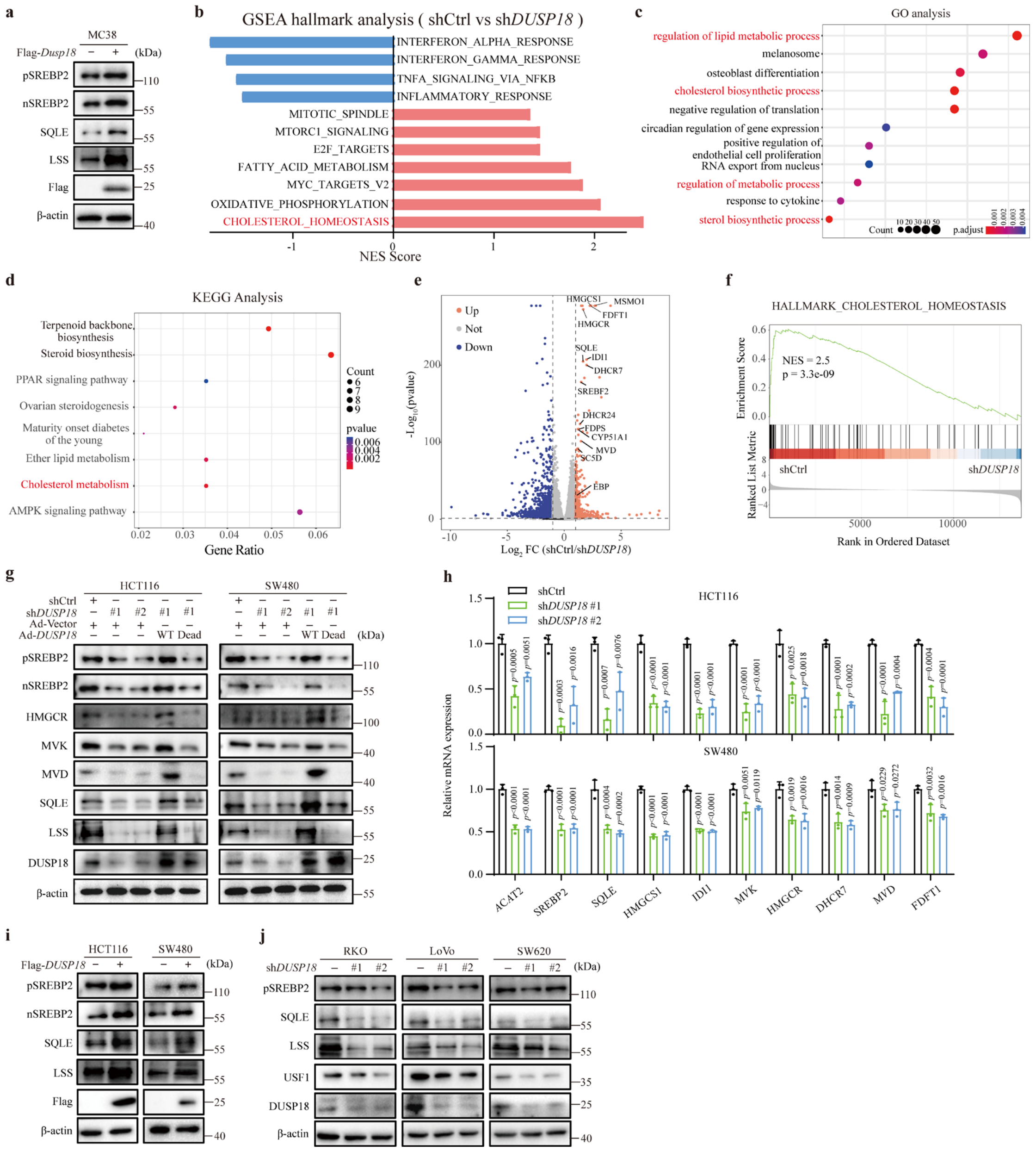

Supplementary Figure. 3 | RNA-Seq of shCtrl and sh*DUSP18* HCT116 cell validated that *DUSP18* promotes cholesterol biosynthesis, related to Fig. 2. a, Immunoblot (IB) analysis for the indicated proteins in control and *Dusp18* over-expression MC38 cells. **b**, Gene-set enrichment analysis (GSEA) in shCtrl versus sh*DUSP18* cells. n = 3 biologically independent samples per group. NES: normalized enrichment score. **c**, Dot plot showing top 10 terms in Gene Ontology (GO) analysis of up-regulated genes in shCtrl versus sh*DUSP18* HCT116 cells. n = 3 biologically independent samples per group. **d**, Dot plot showing top 10 terms in KEGG analysis of up-regulated genes in shCtrl versus sh*DUSP18* HCT116 cells. **e**, Volcano plot showing differential gene expression for RNA-seq results from shCtrl versus sh*DUSP18* HCT116 cells. Red dots

represent 754 upregulated genes (Log_2 FC > 0.585 and *p* value < 0.05) and dots in blue represent 1303 downregulated genes (Log_2 FC < -0.585 and *p* value < 0.05) in shCtrl versus sh*DUSP18* HCT116 cells. Highlighted genes are involved in cholesterol biosynthesis pathways. **f**, GSEA analysis for cholesterol homeostasis pathway genes in shCtrl versus sh*DUSP18* HCT116 cells. **g, h**, protein levels (**g**) and mRNA expression (**h,** n = 3) of the indicated group in HCT116 cells. **i, j**, protein levels of the indicated group in some human colorectal cancer cell lines. Data are presented as mean ± SD (**h**). *P*-values were calculated by one-way ANOVA (**h**), modified Fisher's exact tests (**c, d**), Kolmogorov–Smirnov tests (**b, f**), Wald-test with Benjamini-Hochberg correction (**e**). ns, not significant. All IB data are representative of three independent experiments. Source data are provided as a Source Data file.

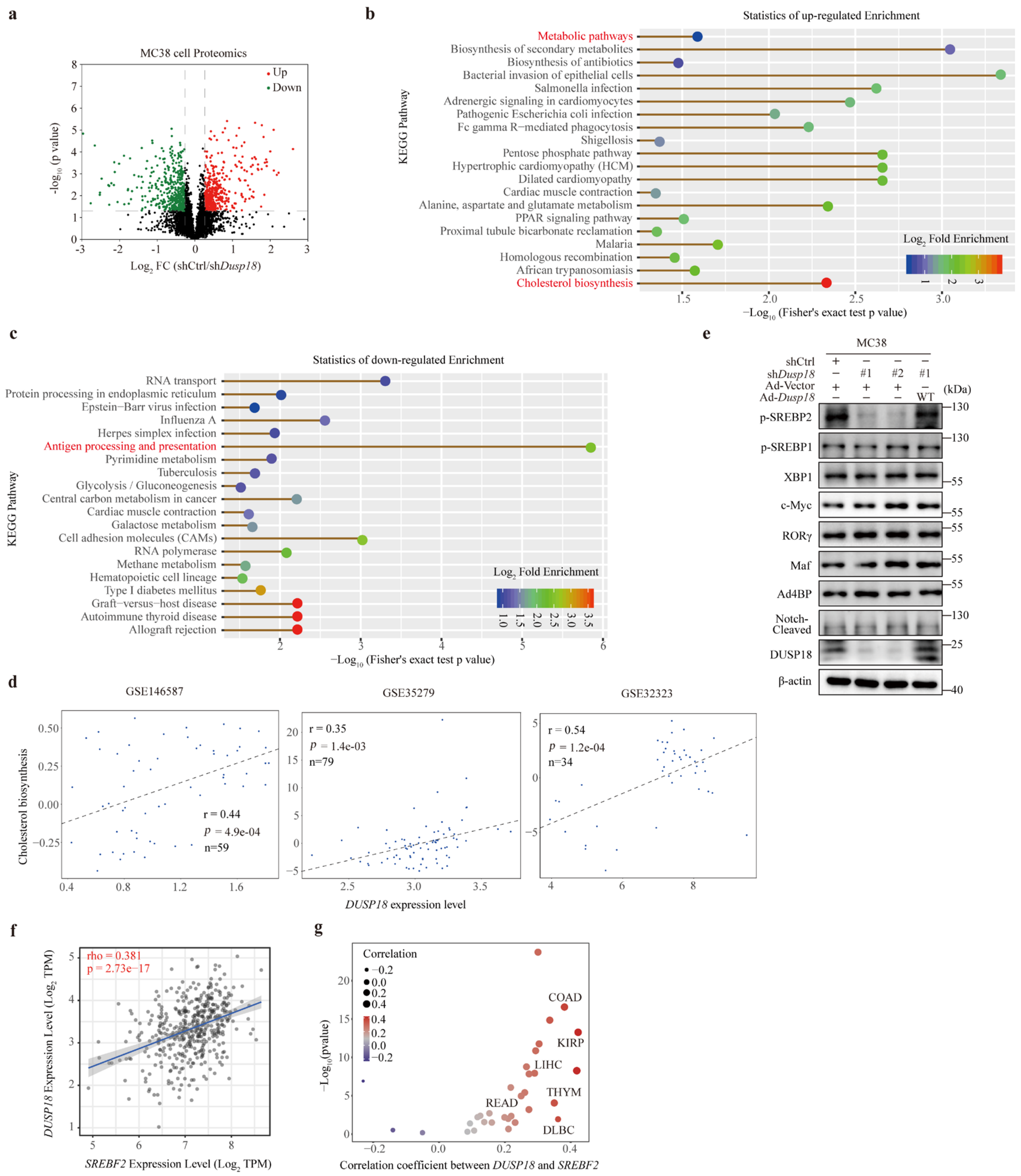

Supplementary Figure. 4 | Proteomics analysis of shCtrl and sh*Dusp18* MC38 cells, related to Fig. 2. a, Volcano plot showing differential protein levels for proteomics results from shCtrl versus sh*Dusp18* MC38 cells. Dots in red represent 449 upregulated proteins (Log_2 FC > 0.585 and p value < 0.05) and dots in green represent 440 downregulated proteins (Log_2 FC < -0.585 and p value < 0.05) in shCtrl versus sh*Dusp18* MC38 cells (n=3). **b**, **c**, A dot map showing top 20 terms in Kyoto Encyclopedia of Genes and Genomes (KEGG) analysis of differential genes in shCtrl versus sh*Dusp18* MC38 cells. n = 3 biologically independent samples per group. **d**, Correlation between *DUSP18* and the cholesterol biosynthesis pathway in three GEO datasets: GSE146587

(n = 59); GSE35279 (n=79); GSE32323 (n=34). **e**, Immunoblot (IB) analysis of indicated protein levels in shCtrl and sh*Dusp18* MC38 cells (n = 2). **f**, Correlation analysis between *DUSP18* mRNA expression and *SREBF2* mRNA expression in TCGA-COAD data (n = 473). **g**, Pan-cancer analysis of the relationship between the *DUSP18* mRNA expression and *SREBF2* mRNA expression in tumor samples. Spearman's rho statistic was used to estimate a rank-based measure of association. *P*-values and R were calculated by Spearman's correlation analysis, two-sided *P*-value was given (**d, f, g**), modified Fisher's exact tests (**b, c**), Wald-test with Benjamini-Hochberg correction (**a**). All IB data are representative of two independent experiments. Source data are provided as a Source Data file.

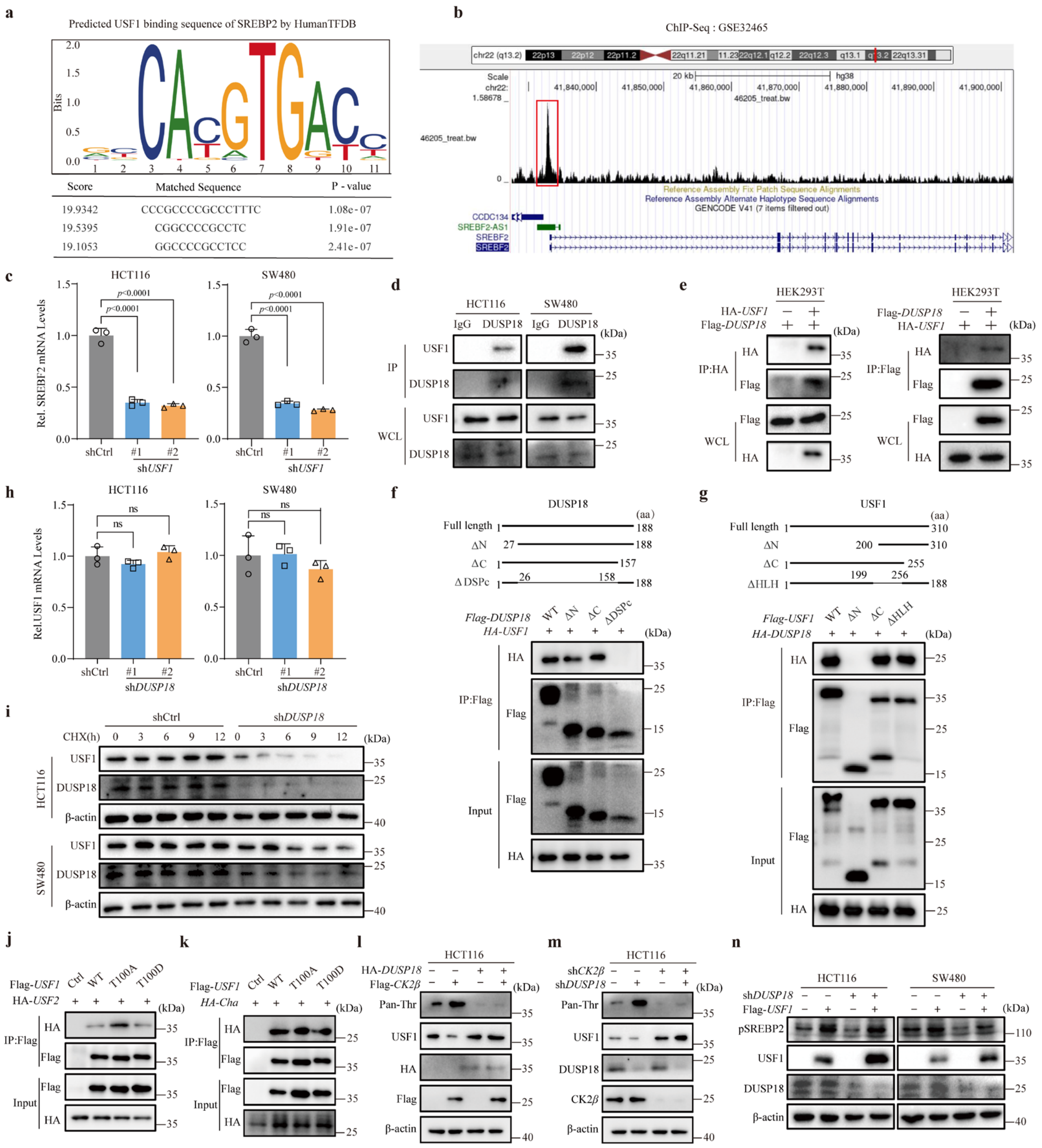

Supplementary Figure. 5 | USF1 is essential for DUSP18's regulatory function, related to Fig. 3. a, Predicted USF1 binding sites in *SREBF2* gene promoter region by Human TFDB website. **b**, The USF1 binding sequence was analyze by CHIP-Seq in the *SREBF2* gene promotor region. **c**, *SREBF2* mRNA levels were measured by qRT-PCR in shCtrl or sh*USF1* cells (n=3). **d**, Endogenous interaction between DUSP18 and USF1 in HCT116 and SW480 cells. Mouse IgG was used as a negative control. **e**, Exogenous interaction between DUSP18 and USF1 in HEK293T cells. **f**, Schematic diagram showing the structure of DUSP18 and its truncation mutants. Flag-*DUSP18* WT or truncation mutants were co-expressed with HA-*USF1* in HEK293T cells. Extracts were immunoprecipitated with anti-Flag affinity agarose and examined by Immunoblot (IB)

analysis. **g**, Flag-*USF1* WT or truncation mutants were co-expressed with HA-*DUSP18* in HEK293 T cells. Extracts were immunoprecipitated with anti-Flag affinity agarose and examined by Immunoblot (IB) analysis. **h**, *USF1* mRNA levels were measured by qRT-PCR in shCtrl or sh*DUSP18* cells. **i**, HCT116 and SW480 cells were transfected with the indicated vectors. Cells were treated with CHX (50 μg/mL) for the indicated time and the expression of USF1 was analyzed by Immunoblot (IB) analysis. **j, k**, Flag-*USF1* WT or mutants were co-expressed with HA-*USF2* (**j**) and HA-*Cha* (**k**) in HEK293 T cells. Extracts were immunoprecipitated with anti-Flag affinity agarose and examined by Immunoblot (IB) analysis. **l**, DUSP18 antagonizes CK2β-mediated threonine phosphorylation of USF1. **m**, Inhibition of *DUSP18* enhances threonine phosphorylation of USF1 in HCT116 cells. This was inhibited by *CK2*β co-depletion. **n**, Over-expression of *USF1* enhances SREBP2 levels regardless of shCtrl or sh*DUSP18* HCT116 and SW480 cells. Data are presented as mean ± SD (**c, h**). *P*-values were calculated by one-way ANOVA (**c, h**). ns, not significant. All IB data are representative of two independent experiments. Source data are provided as a Source Data file.

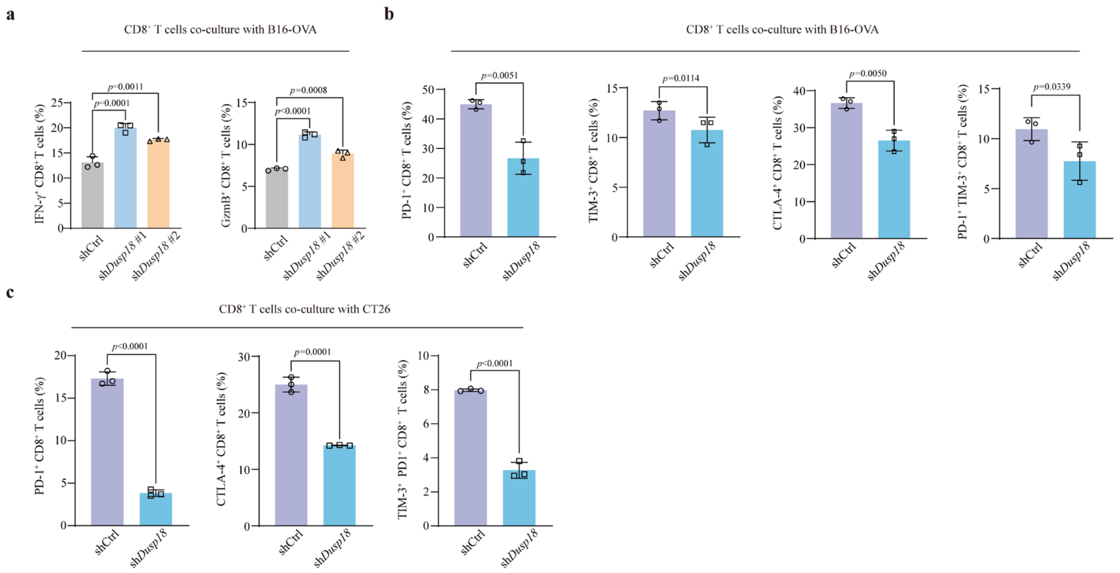

Supplementary Figure. 6 | *DUSP18* inhibition in tumor cells induces T cell activation *in vitro*, related to Fig. 4. a, Quantification of IFN-γ (left) or GzmB (right) production in CD8$^+$ T cells when co-cultured with B16-OVA cells (n = 3). **b**, Quantification of PD-1$^+$, TIM-3$^+$, CTLA-4$^+$ and PD-1$^+$ TIM-3$^+$ percentage in CD8$^+$ T cells when co-cultured with B16-OVA cells (n = 3). **c**, Quantification of PD-1$^+$, CTLA-4$^+$ and PD-1$^+$ TIM-3$^+$ cell percentage in CD8$^+$ T cells when co-cultured with CT26 cells (n = 3). Data are presented as mean ± SD (**a-c**). *P*-values were calculated by unpaired two-tailed t-tests (**b, c**), one-way ANOVA (**a**). ns, not significant. Source data are provided as a Source Data file.

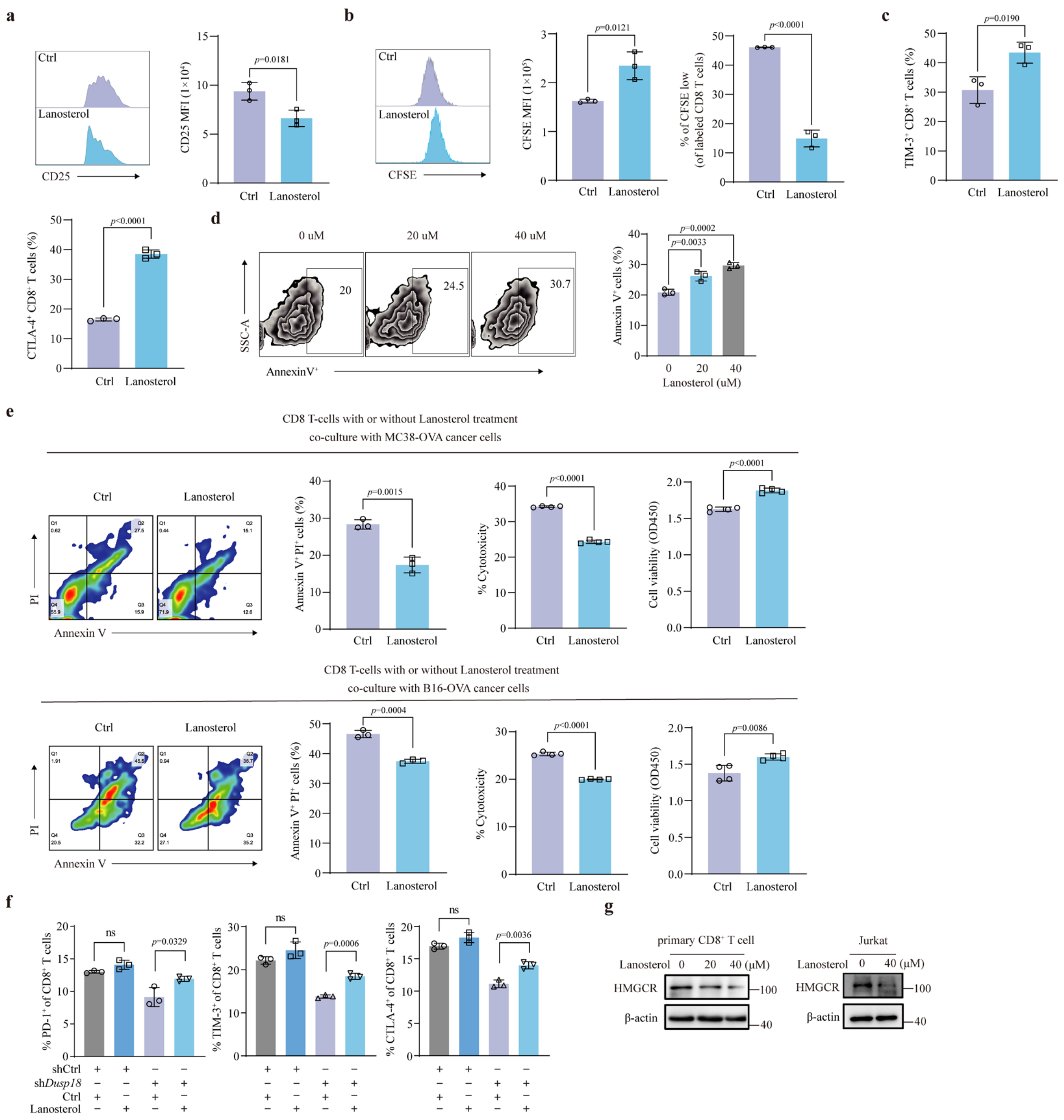

Supplementary Figure. 7 | Tumor-cell-derived Lanosterol promotes CD8⁺ T cell inactivation in TME, related to Fig. 5. a, FACS analysis of CD8⁺ T cell activation marker CD25 expression following lanosterol treatment (n = 3). **b**, FACS analysis of CD8⁺ T cell proliferation following lanosterol treatment (n = 3). **c**, Quantification of TIM-3⁺ and CTLA-4⁺ cell percentage in CD8⁺ T cells after lanosterol treatment (n = 3). **d**, Quantification of apoptotic cell percentage in CD8⁺ T cells following lanosterol treatment (n = 3). **e**, The cytotoxic effect of CD8⁺ T cells was measured by Annexin V / propidium iodide staining, LDH release and cell viability of MC38-OVA and B16-OVA after co-cultured with CD8⁺ T cells treated by Ctrl or lanosterol for 24 h (n = 3). **f**, Quantification of PD-1⁺, TIM-3⁺ and CTLA-4⁺ percentage in CD8⁺ T cells, related to **Fig.**

5j (n = 3). **g**, The indicated protein levels were confirmed by Immunoblot (IB) analysis following the indicated treatment. Data are presented as mean ± SD (**a-f**). *P*-values were calculated by unpaired two-tailed t-tests (**a-c, e, f**), one-way ANOVA (**d**). All IB data are representative of three independent experiments. ns, not significant. Source data are provided as a Source Data file.

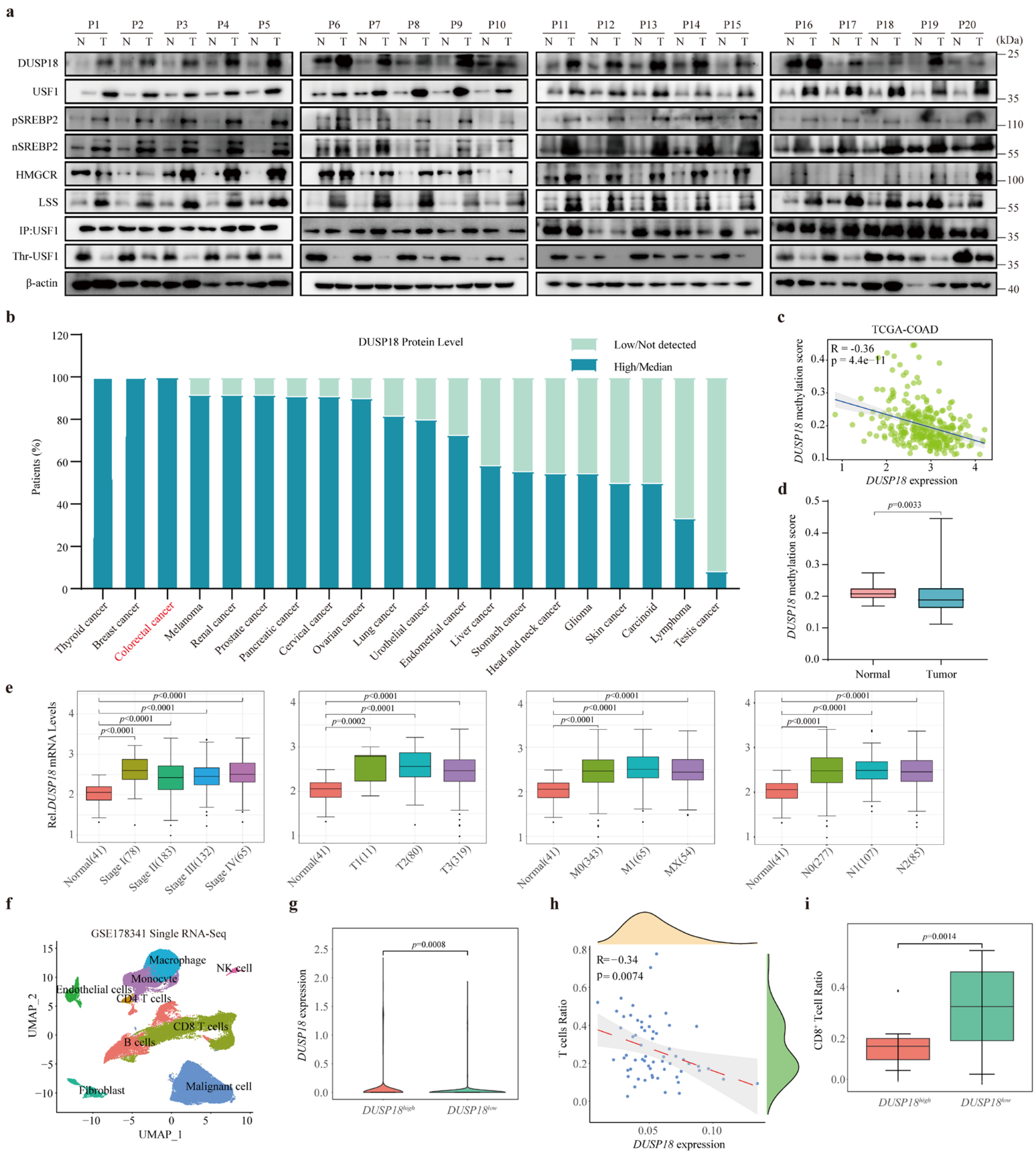

Supplementary Figure. 8 | *DUSP18* is overexpressed in CRC patient tumors and predicts immune deserts, related to Fig. 6. a, Immunoblot (IB) analysis of the indicated proteins in 20 pairs of human CRC samples (T) with adjacent normal colon tissues (N). **b**, DUSP18 protein levels among various human cancers in the Human Protein Atlas database. **c**, Correlation of *DUSP18* mRNA level with its methylation level in CRC tissues from TCGA dataset. Each point is an individual sample (n=352 samples). **d**, Box plot showing methylation level analysis between normal and COAD samples (normal samples: n=38; tumor samples: n=314). **e**, Relative *DUSP18* mRNA expression grouped by clinical stage, T stage (T), distal metastases (M), lymph node involvement (N), in CRC samples from TCGA database. **f**, Identification of TME cell populations.

Uniform manifold approximation and projection (UMAP) embeddings of single-cell RNA-seq profiles showing 10 clusters identified by integrated analysis, colored by cluster (n = 30 samples). **g**, Violin plots of *DUSP18* mRNA expression in *DUSP18* high (n = 15 samples) and low (n = 15 samples) CRC patients. **h**, The correlation between *DUSP18* mRNA expression and T cell infiltration in the GSE178341 cohort (n = 30 samples). **i,** Quantification of $CD8^+$ T cell percentage in the GSE178341 cohort between the $DUSP18^{high}$ (n = 2264 cells) and $DUSP18^{low}$ (n = 2804 cells) groups. Data are presented as mean ± SD (**d, e, g, i**). *P*-values were calculated by unpaired two-tailed t-tests (**g i**), one-way ANOVA (**e**), Mann-Whitney test (**d**). *P*-values and R were calculated by Spearman's correlation analysis, two-sided *P*-value was given (**c, h**). ns, not significant. Source data are provided as a Source Data file.

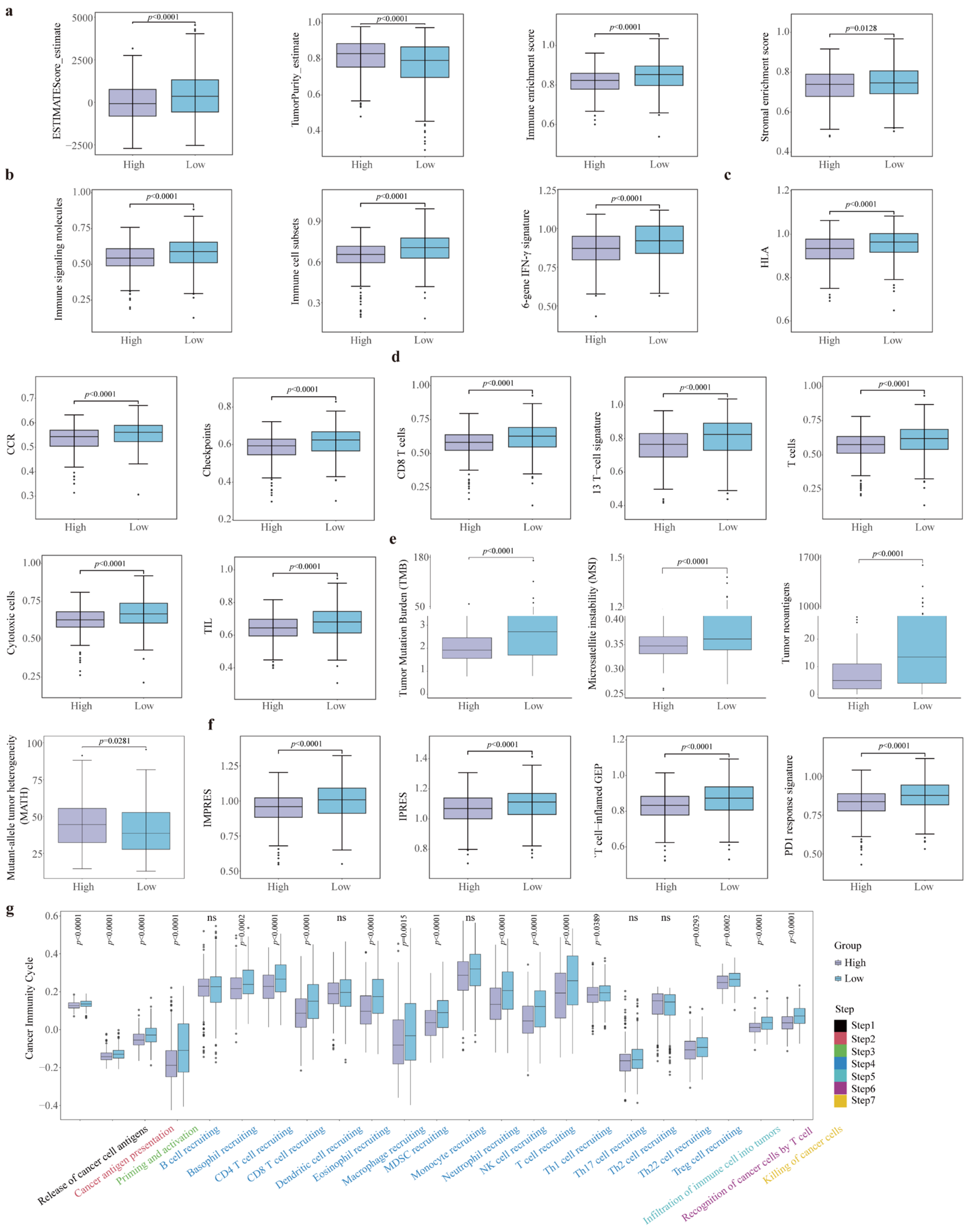

Supplementary Figure. 9 | *DUSP18* is negatively correlated with immune-related signaling, related to Fig. 6. a-d, Previously reported immune-related gene signatures, calculated using the single-sample gene-set enrichment analysis (ssGSEA) algorithm, were used to characterize immune signaling based on *DUSP18* mRNA expression (online Supplemental Data 6) (*DUSP18*^{high} samples=320, *DUSP18*^{low} samples=320). **e**, Tumor mutation burden (TMB) (*DUSP18*^{high} samples=141, *DUSP18*^{low} samples=141),

Microsatellite Instability (MSI) (*DUSP18*^{high} samples=149, *DUSP18*^{low} samples=136), Tumor neoantigen (*DUSP18*^{high} samples=122, *DUSP18*^{low} samples=148) and Mutant-allele tumor heterogeneity (MATH) (*DUSP18*^{high} samples=186, *DUSP18*^{low} samples=186) were identified by Sangerbox website (http://sangerbox.com/) grouped by *DUSP18* mRNA expression. **f**, T cell inflamed gene expression profile (GEP), innate anti-PD-1 resistance (IPRES), immuno-predictive score (IMPRES) and PD1 response signatures were higher in *DUSP18*-low groups (*DUSP18*^{high} samples=320, *DUSP18*^{low} samples=320). **g**, Differences in the various steps of the cancer immunity cycle between high-*DUSP18* and low-*DUSP18* groups in TCGA-COAD cohort. Release of cancer cell antigens (step 1); cancer antigen presentation (step 2); priming and activation (step 3); trafficking of immune cells to tumors (step 4) (mainly those that exert antitumor immunity); immune cell infiltration (step 5); recognition of cancer cells by T cell (step 6); killing of cancer cell (step 7) (*DUSP18*^{high} samples=320, *DUSP18*^{low} samples=320). Data are presented as mean ± SD (**a-g**). *P*-values were calculated by Mann-Whitney test (**a-g**). ns, not significant. Source data are provided as a Source Data file.

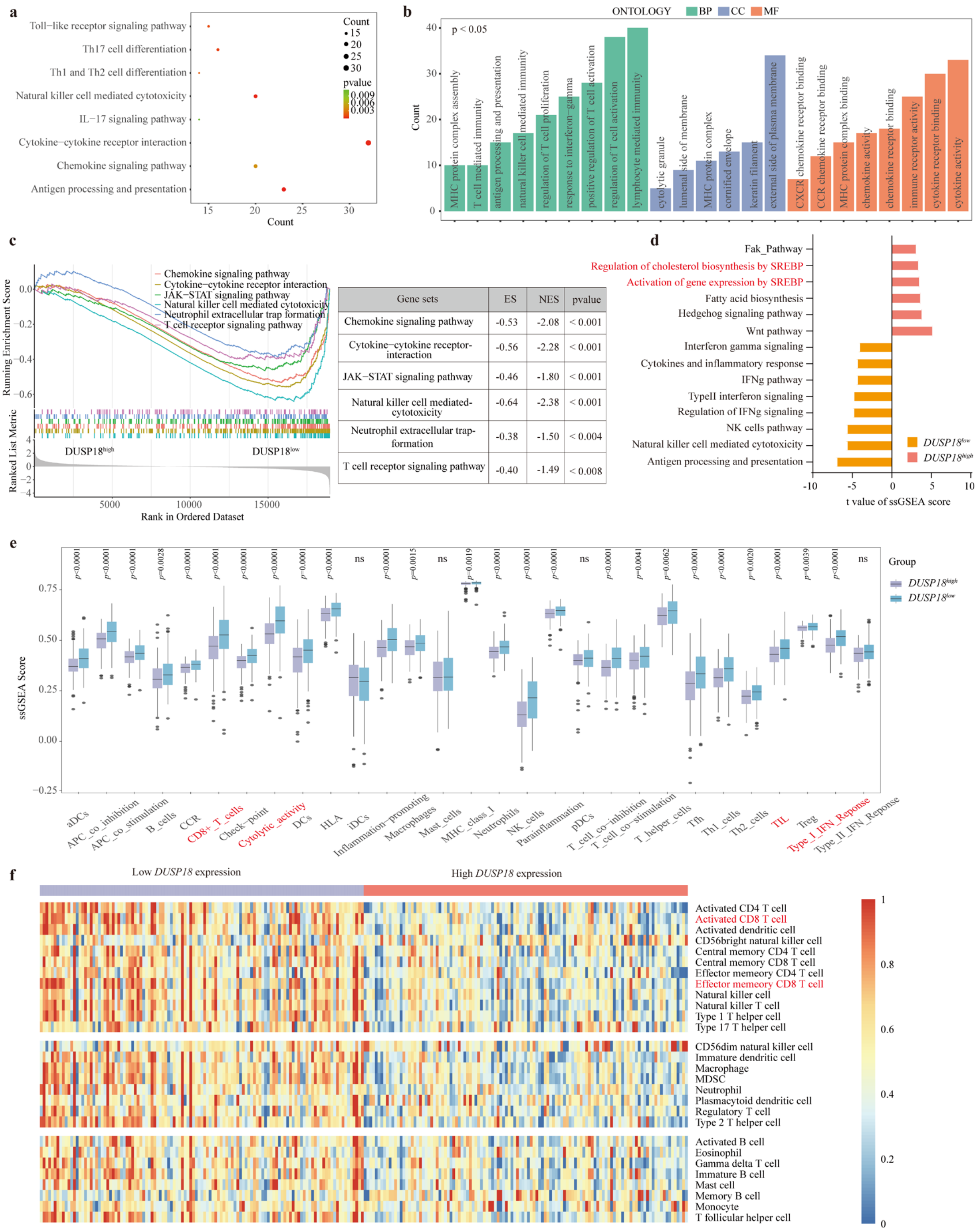

Supplementary Figure. 10 | *DUSP18* is negatively correlated with CD8^{+} T cell activation, recruitment signaling, related to Fig. 6. a-b, KEGG (**a**) and GO (**b**) analysis b, based on the down-regulated genes that were screened between *DUSP18* high (n = 118) vs low (n = 118) mRNA expression separated by interquartile range of *DUSP18* expression in the COAD cohort (n = 473). **c**, Significant functional gene sets enriched were identified in the COAD cohort using GSEA (n = 473). **d**, Enrichment of different gene signature scores between *DUSP18* high (n = 118) vs low (n = 118) mRNA

expression levels in the COAD cohort using GSEA. **e**, Bar plot representation showing the correlation between *DUSP18* high (n = 118) vs low (n = 118) mRNA expression levels and 29 immune-related signaling in the COAD cohort. **f**, Heatmap representation showing the correlation between *DUSP18* high (n = 118) vs low (n = 118) mRNA expression levels and 28 immune cells in the COAD cohort. Data are presented as mean ± SD (**e**). *P*-values were calculated by Mann-Whitney test (**e**), modified Fisher's exact tests (**a, b**), Kolmogorov–Smirnov tests(**c**). ns, not significant. Source data are provided as a Source Data file.

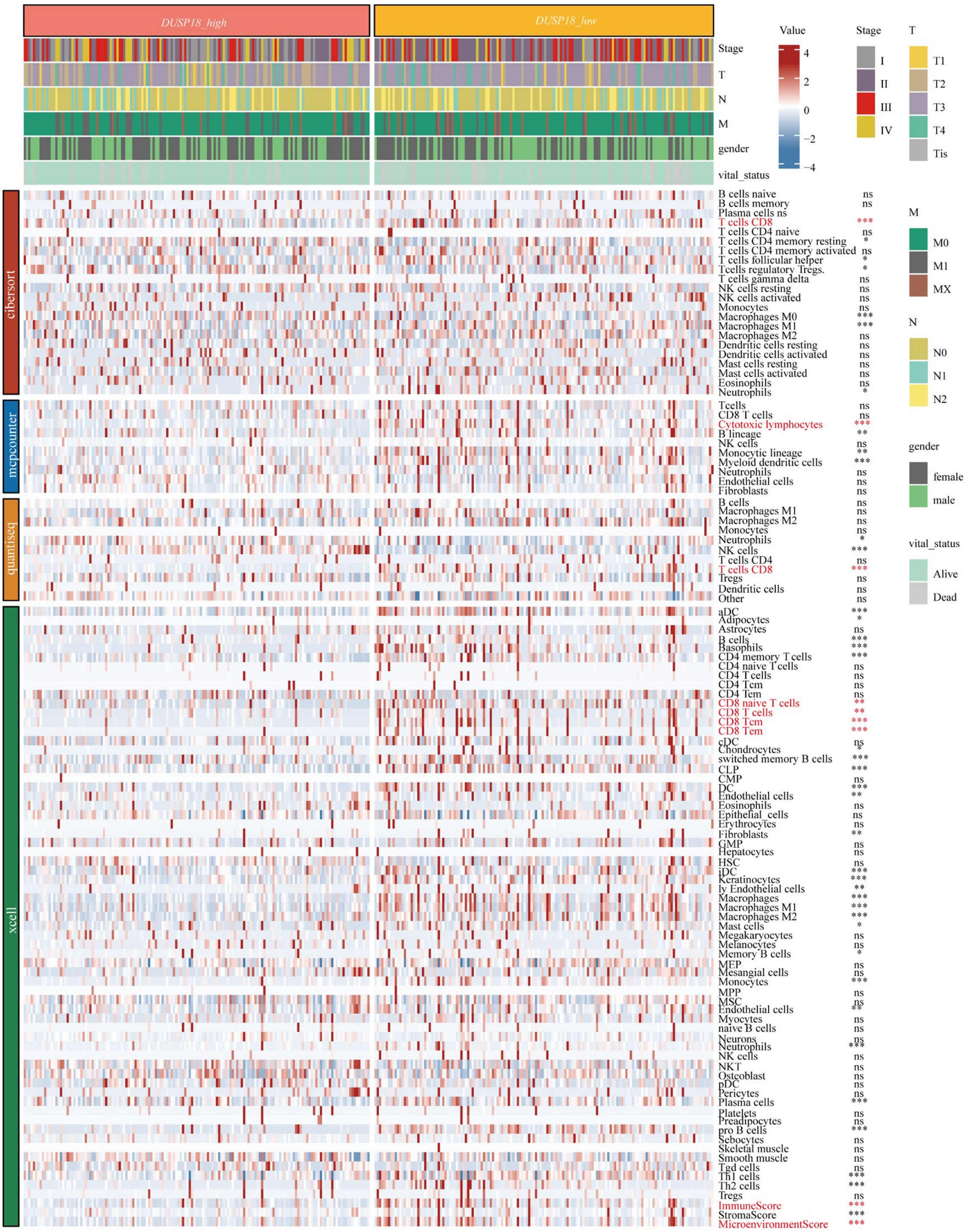

Supplementary Figure. 11 | Analysis of *DUSP18* mRNA expression in relation to the COAD immune landscape, related to Fig. 6. The relationship between *DUSP18* high (n = 118) vs low (n = 118) mRNA expression separated by interquartile range of *DUSP18* expression in the COAD cohort (n = 473) and clinical characterization was analyzed, and its association with immune infiltration was analyzed using four algorithms. *P*-values were calculated by Mann-Whitney test. $^{**}P < 0.05$, $^{**}P < 0.01$, $^{***}P < 0.001$, $^{****}P$

< 0.0001. Source data and exact p-value are provided as a Source Data file.

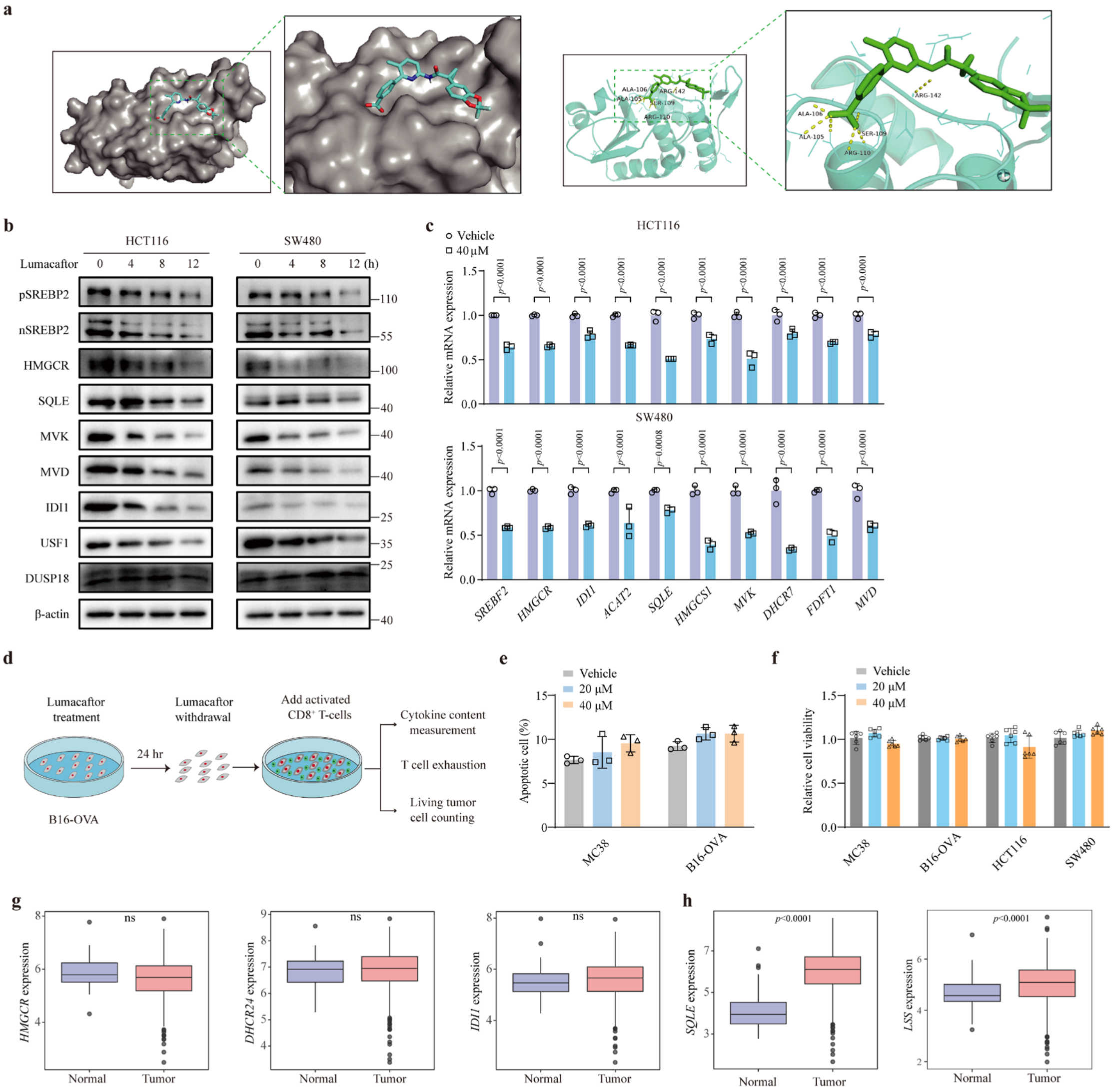

Supplementary Figure. 12 | Identification and characterization of Lumacaftor as a potent DUSP18 inhibitor, related to Fig. 7. a, Molecular docking of Lumacaftor and DUSP18 is shown based on the crystal structure of DUSP18 (PDB code: 2esb), and the bound residues are shown. **b**, Immunoblot (IB) analysis of the indicated proteins in HCT116 and SW480 cell lines following exposure to 40 μM Lumacaftor for 24 h. **c**, mRNA levels of indicated genes from HCT116 and SW480 cell treated by Ctrl or 40 μM Lumacaftor for 24h were analyzed using RT-qPCR (n = 3). **d**, Graphical overview of the method to assess T cell activity. **e**, Percentage of apoptotic cells in MC38 and B16-OVA tumor with or without Lumacaftor treatment for 24 h. Analysis was by flow cytometry (n = 3). **f**, Effect of Lumacaftor on cell viability measured by CCK-8 assay after 72 h of treatment (n = 5). **g, h**, Transcriptional levels of indicated genes in CRC tumors and

normal tissues from TCGA-COAD (normal sample=41, tumor sample=473). Data are presented as mean ± SD (**c, e-h**). *P*-values were calculated by unpaired two-tailed t-tests (**c**), one-way ANOVA (**e, f**), Mann-Whitney test (**g, h**). ns, not significant. All IB data are representative of two independent experiments. Source data are provided as a Source Data file.

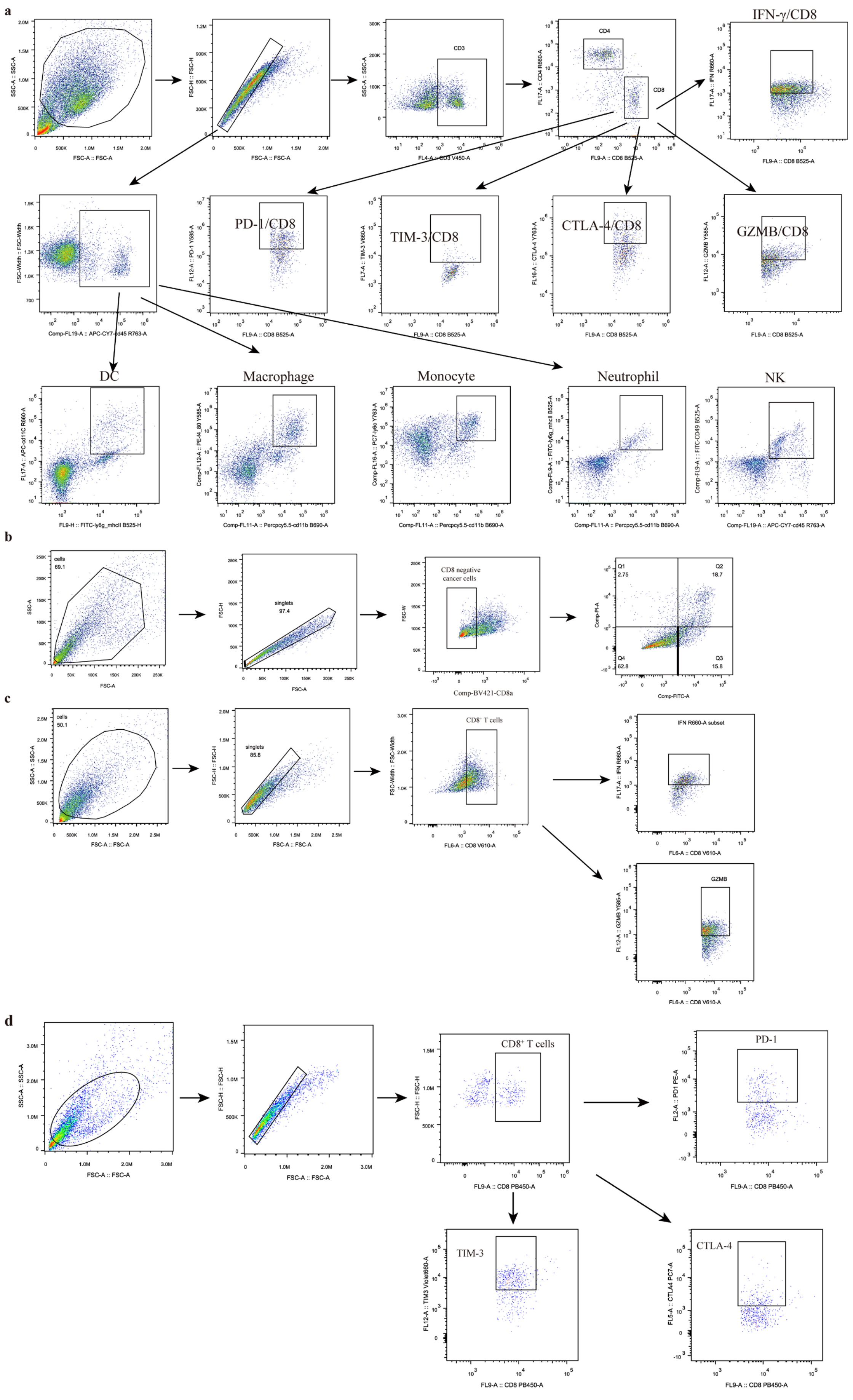
a
IFN-γ/CD8
CD3
CD4
CD8
PD-1/CD8
TIM-3/CD8
CTLA-4/CD8
GZMB/CD8
DC
Macrophage
Monocyte
Neutrophil
NK
b
cells
69.1
singlets
97.4
CD8 negative cancer cells
Q1
2.75
Q2
18.7
Q4
62.8
Q3
15.8
Comp-BV421-CD8a
Comp-FITC-A
c
cells
50.1
singlets
85.8
CD8+ T cells
IFN R660-A subset
GZMB
d
CD8+ T cells
PD-1
TIM-3
CTLA-4

e

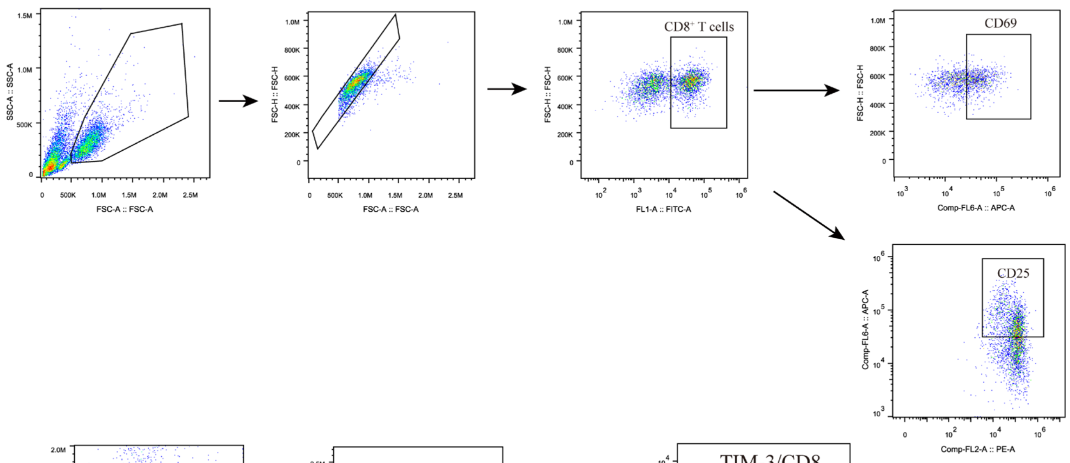

f

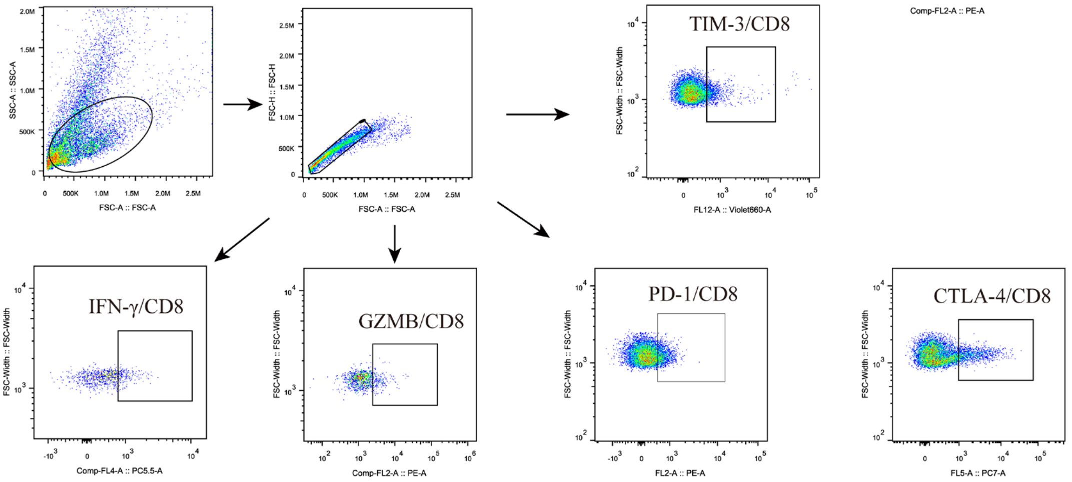

Supplementary Figure. 13 | Gating and sorting strategies. a, Representative flow cytometry gating and sorting strategies of tumor-infiltrating immune cells and CD8⁺ T cell related phenotypes in CRC mouse model (Fig. 1n-p, 3m-o, 5k, 8d-g, 8k-m, Supplementary Figure. 1o-p, 2b, 2f-h and 7f). **b,** Representative flow cytometry gating strategies of apoptotic tumor cells when co-cultured with mouse CD8 T-cells for T-cell killing assay (Fig. 4f-g, 7i and Supplementary Figure. 7e). **c,** Representative flow cytometry gating strategies of IFNγ⁺ CD8+ T cells and GzmB⁺ CD8⁺ T cells co-cultured with MC38 or B16 cells (Fig.4j, 7k, Supplementary Figure. 6a). **d,** Representative flow cytometry gating strategies of PD-1⁺ CD8⁺ T cells, TIM-3⁺ CD8⁺ T cells, and CTLA-4⁺ CD8⁺ T cells co-cultured with MC38 or B16 cells (Fig.4k-l, 7j, Supplementary Figure. 6b-c). **e,** Representative flow cytometry gating strategies of CD69⁺ CD8⁺ T cells and CD25⁺ CD8⁺ T cells co-cultured with MC38 cells (Fig. 4i). **f,** Representative flow cytometry gating strategies of CD8⁺ T cell-associated effector and exhaustion molecules when treated by lanosterol (Fig. 4e-i, 5m-n, Supplementary Figure. 7a, 7c-d).

Supplementary Table 1. shRNA sequences.

shRNA	Target Sequences (5'>3')
sh*DUSP18* #1	GCTTCTTACAGTGAGTGCCAA
sh*DUSP18* #2	GCCTCGCCTACCTCATGAAGT
sh*Dusp18* #1	CCTACTGTCCAGCAATCAGAT
sh*Dusp18* #2	GCTCCTACTGTCCAGCAATCA
sh*USF1* #1	GCCAGAGTAAAGGTGGGATTC
sh*USF1* #2	GCCGAGACAAGATCAACAACT
sh*Kras*	CTATACATTAGTCCGAGAAAT

Supplementary Table 2. Primers used for constructs.

Insert	Forward Primer (5'>3')	Reverse Primer (5'>3')
HA-*DUSP18*	CGCGTCGACATGACAGCACCCTCGTGTG	CGCGCGGCCGCTCACAGTGGAATCATCAAACGGACT
Flag-*DUSP18*	CGCGTCGACATGACAGCACCCTCGTGTG	CGCGCGGCCGCTCACAGTGGAATCATCAAACGGACT
Flag-*DUSP18*-△N	CGCTCTAGAATGCTGTATATCAGCAATGGTGTGG	CGCGCGGCCGCTCACAGTGGAATCATCAAACGGACT
Flag-*DUSP18*-△C	CGCGTCGACATGACAGCACCCTCGTGTG	CGCGAATTCTTGGAACTCATAGTGGATGAGC
Flag-*DUSP18*-△DSPc	TTGTTTGGCAAGAACACTGTGCAC	GTGCACAGTGTTCTTGCCAAACAAGCTTTTGGTTATCTGCGAGAG
Flag-*USF1*	CGCGTCGACATGAAGGGGCAGCAGAAAACAGC	CGCGCGGCCGCTTAGTTGCTGTCATTCTTGATGACGACC
Flag-*USF1*-△N	CGCGTCGACCGCAGGGCTCAGCATAATG	CGCGCGGCCGCTTAGTTGCTGTCATTCTTGATGACGACC
Flag-*USF1*-△C	CGCGTCGACATGAAGGGGCAGCAGAAAACAGC	CGCGCGGCCGCTTACCGAAGCTCCTGGATATAATCAC
Flag-*USF1*-△HLH	CAGAGTAACCACCGCTTG	CTTCAGACAAGCGGTGGTTACTCTGTTTCTCATCCCGAGTCGTCCG
HA-*USF1*	CGCGTCGACATGAAGGGGCAGCAGAAAACAGC	CGCGCGGCCGCTTAGTTGCTGTCATTCTTGATGACGACC
HA-*CK2*	CGCGTCGACATGTCGGGACCCGTGCC	CGCGCGGCCGCTTACTGCTGAGCGCCAGCG
HA-*USF2*	CGCGTCGACATGGACATGCTGGACCCGG	CGCGCGGCCGCTCACTGCCGGGTGCCCTC

Supplementary Table 3. Chemical reagents and kits.

Reagent or kit	Source	Identifier
Lanosterol	MedChemExpress	HY-W020033
Cycloheximide	MedChemExpress	HY-12320
Ro 48-8071	MedChemExpress	HY-18630A
CFSE	MedChemExpress	HY-D0056
MG132	MedChemExpress	HY-13259
Lumacaftor	MedChemExpress	HY-13262
MTT	Sigma-Aldrich	M5655
AOM	Sigma-Aldrich	A5486
DSS	MP Biomedicals	02160110-CF
Trizol	Invitrogen	10296010
SYBR Green master mix	Abclonal	RK21203
Percoll	solarbio	P8370
Phorbol 12-myristate 13-acetate	MedChemExpress	HY-18739
Ionomycin	MedChemExpress	HY-13434
IL-2	MedChemExpress	HY-P7077
HiScript III 1st Strand cDNA Synthesis Kit	Vazyme	R312-01
Intracellular Fixation & Permeabilization Buffer	eBioscience	88-8823-88

Supplementary Table 4. Antibodies used in this study.

Antibody	Source	Identifier (dilution)
DUSP18	Santa Cruz	Cat# sc-376923 (1:500)
USF1	Abclonal	Cat# A20903 (1:2000)
SREBP2	Proteintech	Cat# 28212-1-AP; RRID: AB_2881091 (1:2000)
HMGCR	Abclonal	Cat# A1633; RRID: AB_2763691 (1:2000)
SQLE	Abclonal	Cat# A2428; RRID: AB_2764343 (1:2000)
Flag	ABclonal	Cat# AE092; RRID: AB_2940847 (1:4000)
HA	Abclonal	Cat# AE008; RRID: AB_2770404 (1:4000)
LSS	Proteintech	Cat# 13715-1-AP; RRID: AB_10597096 (1:1000)
β-actin	Proteintech	Cat# 66009-1-Ig; RRID: AB_2687938 (1:4000)
Myc-tag	Proteintech	Cat# 16286-1-AP; RRID: AB_11182162 (1:2000)
AKT	Abclonal	Cat# A17909; RRID: AB_2861754 (1:2000)
p-AKT	Abclonal	Cat# AP1208; RRID: AB_3076455 (1:2000)
ERK1/2	Proteintech	Cat# 11257-1-AP; RRID: AB_2139822 (1:2000)
p-ERK1/2	Proteintech	Cat# 28733-1-AP; RRID: AB_2881202 (1:2000)
ELK1	Proteintech	Cat# 27420-1-AP; RRID: AB_2880867 (1:2000)
p-ELK1	Abclonal	Cat# AP0033; RRID: AB_2771079 (1:2000)
Caspase-3	Proteintech	Cat# 66470-2-Ig; RRID: AB_2876892 (1:2000)
c-Myc	Abclonal	Cat# A15240; RRID: AB_2762136 (1:2000)
IDI1	Abclonal	Cat# A13826 (1:2000)
GZMB	Abclonal	Cat# A2557; RRID: AB_2764445 (1:2000)
IFN gamma	Abclonal	Cat# A12450; RRID: AB_2759294 (1:2000)
HRP-conjugated anti-rabbit	Bio-Rad	Cat# 170-6515 (1:5000)
HRP-conjugated anti-mouse	Bio-Rad	Cat# 170-6516 (1:5000)
Goat Anti-Mouse IgG Heavy Chain	Abclonal	Cat# AS064; RRID: AB_2864058 (1:4000)

Goat Anti-Mouse IgG Light Chain	Abclonal	Cat# AS062; RRID: AB_2864056 (1:4000)
APC anti-mouse CD69 Antibody	Biolegend	Cat# 104513; RRID: AB_492844 (1:50)
PE anti-mouse CD25	eBioscience	Cat# 12-0251-81; RRID: AB_465606 (1:50)
FITC anti-mouse I-Ab	Biolegend	Cat# 116405; RRID: AB_313724 (1:50)
APC anti-mouse CD11c	Biolegend	Cat# 117309; RRID: AB_313778 (1:50)
FITC anti-mouse Ly-6G	Biolegend	Cat# 127605; RRID: AB_1236488 (1:50)
PerCP/Cyanine5.5 anti-mouse/human CD11b	Biolegend	Cat# 101227; RRID: AB_893233 (1:50)
PE/Cyanine7 anti-mouse Ly-6C	Biolegend	Cat# 128017; RRID: AB_1732093 (1:50)
PE anti-mouse F4/80 Antibody	Biolegend	Cat# 123109; RRID: AB_893498 (1:50)
FITC anti-mouse CD8a	Biolegend	Cat# 100705; RRID: AB_312744 (1:50)
APC anti-mouse CD4	Biolegend	Cat# 100515; RRID: AB_312718 (1:50)
APC/Cyanine7 anti-mouse CD45	Biolegend	Cat# 157618; RRID: AB_2890720 (1:50)
APC anti-mouse IFN-gamma-	eBioscience	Cat# 17-7311-82; RRID: AB_469504 (1:50)
PE- anti-mouse Granzyme B	eBioscience	Cat# 12-8898-82; RRID: AB_10870787 (1:50)
APC-MHC Class I (H-2Kb)	eBioscience	Cat# 17-5958-80; RRID: AB_1311283 (1:50)
APC- OVA257-264 (SIINFEKL) peptide bound to H-2Kb -	eBioscience	Cat# 17-5743-82; RRID: AB_1311286 (1:50)
eFluor™ 450-CD8a	eBioscience	Cat# 48-0081-82; RRID: AB_1272198 (1:50)
PE-CD279 (PD-1) -	eBioscience	Cat# 12-9985-82; RRID: AB_466295 (1:50)
BD OptiBuild™ BV650 Mouse Anti-Mouse CD366 (TIM-3)	BD Pharmingen	Cat# 747623; RRID: AB_2744189 (1:50)
PE/Cyanine7 anti-mouse CD152-	Biolegend	Cat# 106313; RRID: AB_2564237 (1:50)
BD Horizon™ BV421 Hamster Anti-Mouse CD3e	BD Pharmingen	Cat# 562600; RRID: AB_11153670 (1:50)
InVivoMAb anti-mouse CD8a	Bio X Cell	Cat# BE0117; RRID: AB_10950145 (100µg/mice)
InVivoMAb anti-mouse PD-1	Bio X Cell	Cat# BE0273; RRID: AB_2687796 (200µg/mice)

Supplementary Table 5. Mouse genotypes primers.

Gene	Forward Primer (5'>3')	Reverse Primer (5'>3')
Dusp18-cko	CTGAGAGGATAAGTTAGGCCATGA	TTCAGGAGGTAGAAGAGAAAGGTG
Villin-Cre	GTGTTTGGTTTGGTTTCCTCTGCATAAGA	GCAGGCAAATTTTGGTGTACGGTCA

Supplementary Table 6. qPCR primers.

Gene	Forward Primer (5'>3')	Reverse Primer (5'>3')
Srebf2	GCAGCAACGGGACCATTCT	CCCCATGACTAAGTCCTTCAACT
Hmgcr	TTGGCCTCCATTGAGATCCG	ACCGCGTTATCGTCAGGATG
Mvd	TAGTCCACCGCTTCAACACC	CCGACCTGAGTGGCAATGAT
Sqle	TGGGCCAAGTCACCTGAATC	CTGGAGAGAACTGCTGCCAA
Dhcr7	AGCTTCAGGCAGGCACTTAG	TGCTGGGATTTCGAAGCCAT
Acat2	CCCGTGGTCATCGTCTCAG	GGACAGGGCACCATTGAAGG
Hmgcs1	AACTGGTGCAGAAATCTCTAGC	GGTTGAATAGCTCAGAACTAGCC
Idi1	ACCAGCCATCTTGATGAAAAACA	CAGCAACTATTGGTGAAACAACC
CHIP-P1	GGTGCTGTGGGAAAAAGGGGAAA	AGGGATGGATTAGACTGGGTAACC
CHIP-P2	CCTCTCTCCCTCCCTTTACTGCC	TGCAGTTGGTAGCGCTATAGTGAC
CHIP-P3	GGGCTGTCCTGTGTGTTGTAGG	TCCCTCCCAGTGGACATCTGG
CHIP-P4	AATGTGCTCCCAGCCAGGC	AGTGAGGTCCCTCCCCCC
CHIP-P5	AACAAACAGGGGGGCGC	CCTGCCGTCCCGCTCAG
Dusp18	TACAGAATCAGTCGCGGGGT	TGTGCCCACTGAAACCTCCTT
Usf1	CTGAAACCGAAGAGGGAACAG	GTTGGGGTCAGGAAAAGTGG
SREBF2	AACGGTCATTCACCCAGGTC	GGCTGAAGAATAGGAGTTGCC
HMGCS1	GATGTGGGAATTGTTGCCCTT	ATTGTCTCTGTTCCAACTTCCAG
HMGCR	TGATTGACCTTTCCAGAGCAAG	CTAAAATTGCCATTCCACGAGC
MVK	CATGGCAAGGTAGCACTGG	GATACCAATGTTGGGTAAGCTGA
MVD	CTCCCTGAGCGTCACTCTG	GGTCCTCGGTGAAGTCCTTG
IDI1	TTTCCAGGTTGTTTTACGAATACG	TCCTCAAGCTCGGCTGGAT
SQLE	CGTGCTCCTCTTGGTACCTCAT	CGGTCAAGGCGGAGATTATC
ACAT2	GCGGACCATCATAGGTTCCTT	ACTGGCTTGTCTAACAGGATTCT
FDFT1	TCAGACCAGTCGCAGTTTCG	CTGCGTTGCGCATTTCC
DHCR7	GGCATCCCAGCTCCAACTC	GGGCTCTCTCCAGTTTACAGATGA

ACTB	ATCATGAAGTGTGACGTGGACA	AGGAGCAATGATCTTGATCTTCA
Actb	GGCTGTATTCCCCTCCATCG	CCAGTTGGTAACAATGCCATGT
