# Supplementary material for: Inhibition of DUSP18 impairs cholesterol biosynthesis and promotes anti-tumor immunity in colorectal cancer
Source: Nat Commun. 2024 Jul 12;15:5851. doi: 10.1038/s41467-024-50138-x (PMC11239938; doi:10.1038/s41467-024-50138-x)
Supplement: Supplementary file 3 — Description of Additional Supplementary Files [file 41467_2024_50138_MOESM3_ESM.pdf]

Description of Additional Supplementary Files

Supplementary Data 1. KPD sub-library information.

Supplementary Data 2. In vivo KPD library screening on MC38 cells.

Supplementary Data 3. Correlation of genome-wide analysis with CTLs.

Supplementary Data 4. MC38 cells Proteomics.

Supplementary Data 5. Metabolomics results.

Supplementary Data 6. Publicly available gene signatures selected in this study.
